# Supplementary material for: Quality and Nutritional Changes of Traditional Cupcakes in the Processing and Storage as a Result of Sunflower Oil Replacements with Refined Olive Pomace Oil
Source: Foods. 2023 May 24;12(11):2125. doi: 10.3390/foods12112125 (PMC10252959; doi:10.3390/foods12112125)
Supplement: Supplementary file 1 [file foods-12-02125-s001.zip › foods-2406112-supplementary.pdf]

SUPPLEMENTARY MATERIAL

Quality and nutritional changes of traditional cupcakes in the processing and storage as a result of sunflower oil replacements with refined olive pomace oil

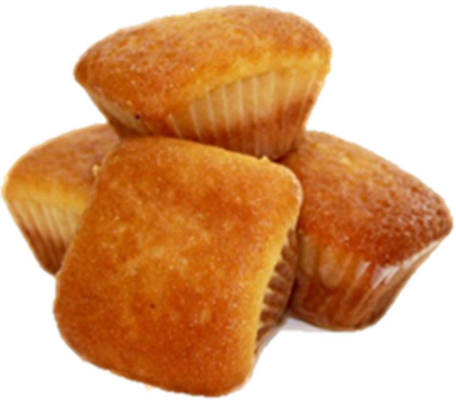

SUPPLEMENTARY METHODS

Method S1 Analysis of volatiles

Hexanal was quantitatively analysed by SPME-GC-MS. One gram of cupcake sample, crumbled by gloved hands, was placed on to a 20-mL vial that was hermetically sealed with a silicone/PTFE septum. The sample was conditioned at 40 ºC for 15 min. A Divinylbenzene/Carboxen/Polydimethylsiloxane (DVB/CAR/PDMS) SPME fibre (Supelco, Bellefonte, PA, USA) was inserted in the vial. The sampling time was 30 min at the same temperature. Then the volatiles were analysed in an Agilent 6890 chromatograph (Agilent Technologies, Inc.) equipped with a split-splitless injector, an Agilent J&W DB-WAX capillary column (60 m × 0.25 mm × 0.25 μm) (Agilent Technologies, Inc.) and a 5975C Mass Spectrometer Detector (Agilent Technologies, Inc.). The injector temperature was 250 ºC and 0.1:1 split ratio was used. The initial oven temperature was 50 ºC (5 min) then it was increased at 4 ºC min^{-1} up to 100 ºC (0 min) and at 17 ºC min^{-1} to 220 ºC (10 min). Hydrogen at 1 mL min^{-1} was the carrier gas. The GC-MS interface operated at 280 ºC, the actual MS source temperature was 230 ºC and that of the MS quadrupole was 150 ºC. The electron impact energy was set at 70 eV and scans in the range 25.00-450.00 a.m.u. were recorded. For quantification purposes, the area under the curve of the total ion current was used and external calibration was applied. Towards this end, an amount of 100 mg of solutions of hexanal in medium chain triacylglycerols (MCT oil), pure C8 MCT oil (Ketosource Ltd., England), in the concentration range of 0.5-20.0 μg g^{-1}, was added to 1 g of fresh SO cupcake sample, previously crumbled. The samples containing hexanal were analysed as described above. A linear calibration curve was obtained for each day of analyses.

Along with hexanal other volatiles were also evaluated on the GC-MS chromatograms. They were tentatively identified using NIST 08 mass spectral library.

SUPPLEMENTARY FIGURES

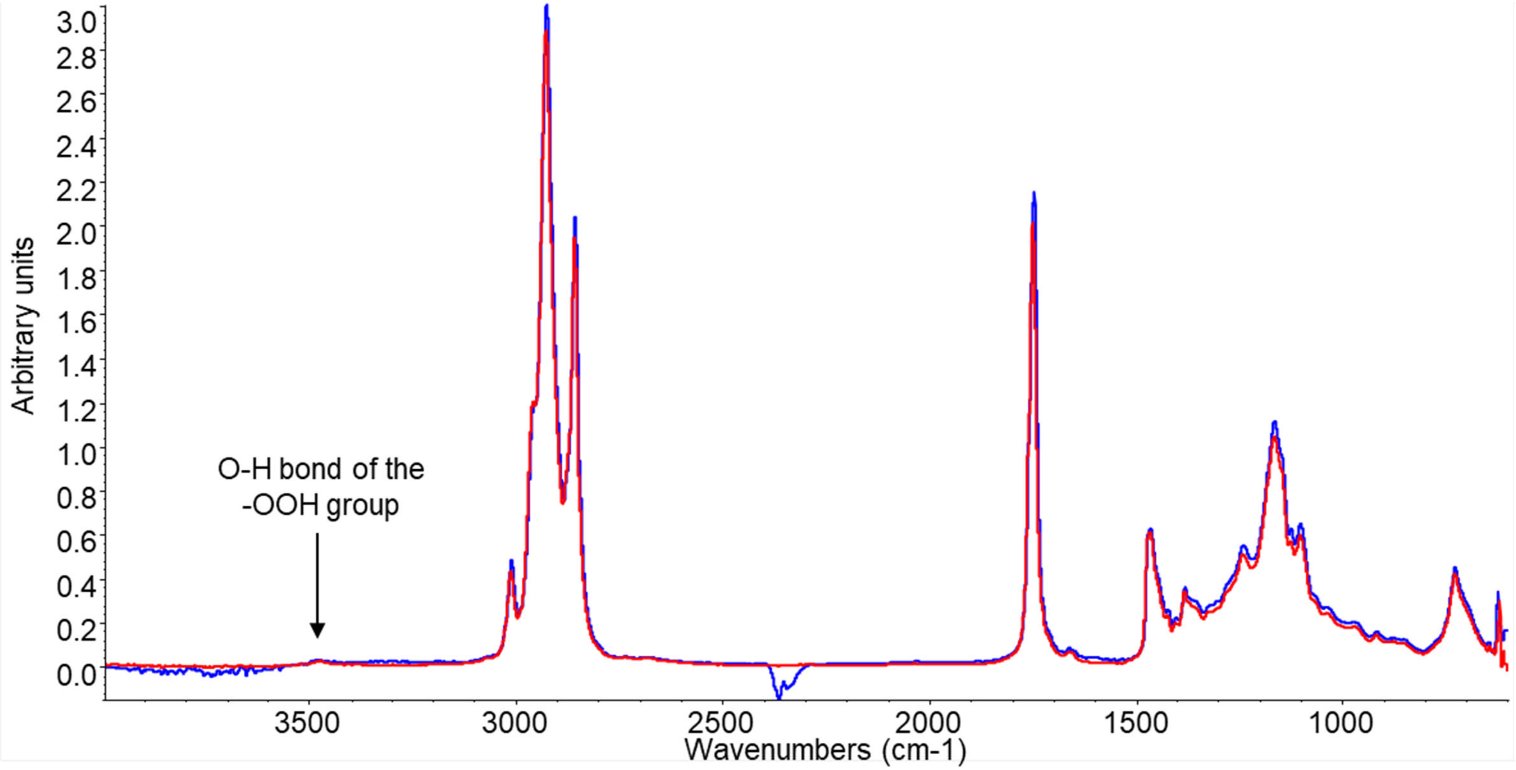

Figure S1 ATR-FTIR spectra of the fat extract obtained from the batter (red) of SO-1 sample and the corresponding sunflower oil (blue).

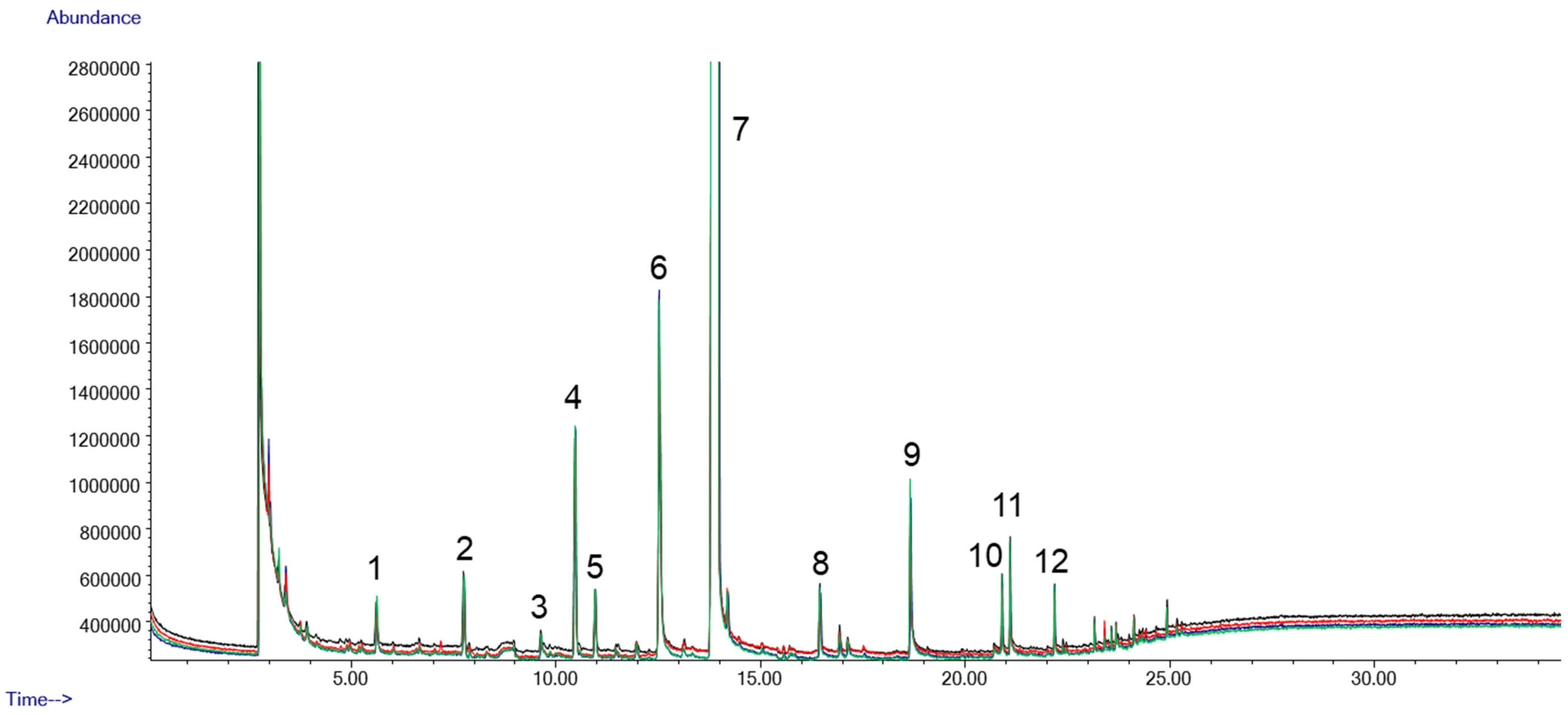

Figure S2 GC-MS chromatograms of fresh samples of SO-1 (black), OPO25-1 (blue), OPO50-1 (green) and OPO-1 (red) cupcakes. Tentatively peak assignation: Ethanol (1), α-pinene (2), unknown compound overlapping with hexanal (3), sabinene (4), 3-carene (5), β-pinene (6), D-limonene (7), 1-methyl-3-isopropylbenzene (8), 6-methyl-5-heptene-2-one (9), 1,5-dimethyl-1,5-cycloctadiene (10), 1-methyl-4-isopropylcyclohexene (11) and 2,6-dimethyl-1,5,7-octatriene (12).

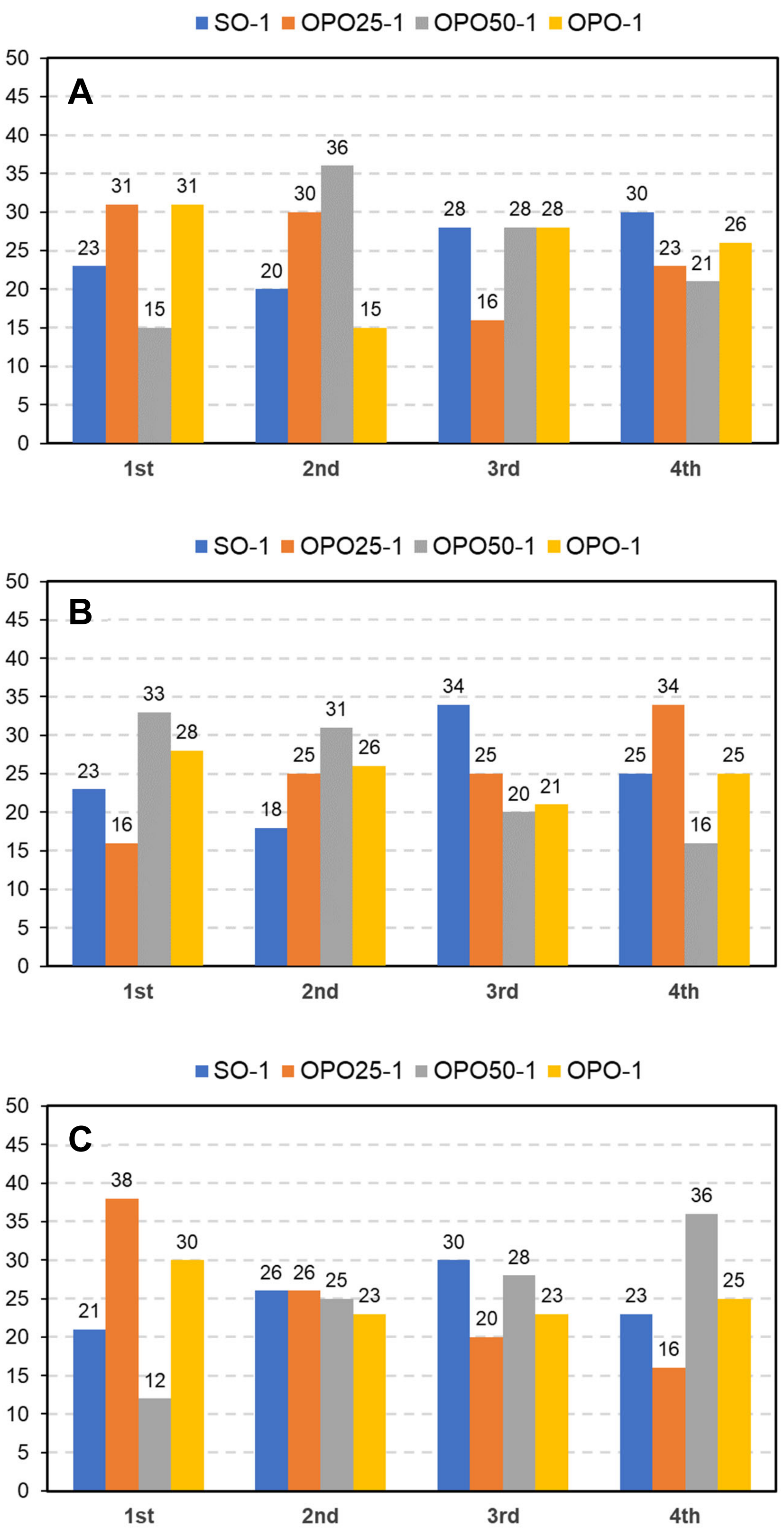

Figure S3 Consumer preference tests on fresh cupcakes (**A**) and cupcakes after 3-month (**B**) and 6-month storage (**C**). Results represent the percentage of panellists (n=61).

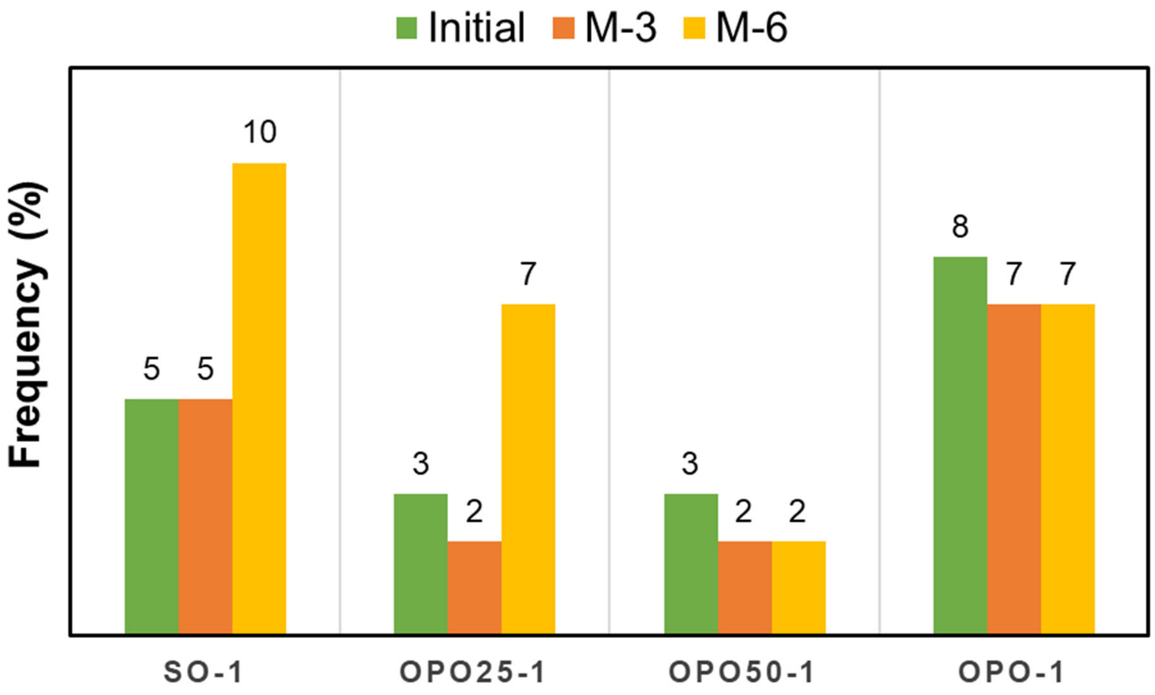

Figure S4 Percentage of consumers indicating rancid odour or taste characteristic of seed oils or nuts in the fresh cakes (Initial), and samples stored for 3 (M-3) and 6 months (6-M). Results represent the percentage of panellists (n=61).

SUPPLEMENTARY TABLES

Table S1 Fatty acid compositions (%) of the oils.

	SO-1	SO-2	SO-3	OPO-1	OPO-2	OPO-3
**C16:0**	6.16 ± 0.02	6.15 ± 0.01	6.38 ± 0.01	12.19 ± 0.01	12.11 ± 0.01	12.50 ± 0.01
**C16:1**	0.12 ± 0.00	0.12 ± 0.00	0.13 ± 0.02	0.93 ± 0.00	0.96 ± 0.01	0.95 ± 0.00
**C18:0**	3.58 ± 0.00	3.57 ± 0.00	3.56 ± 0.01	2.53 ± 0.00	2.58 ± 0.04	2.39 ± 0.01
**C18:1**	37.90 ± 0.14	38.00 ± 0.03	32.31 ± 0.11	71.51 ± 0.02	71.51 ± 0.07	70.51 ± 0.03
**C18:2**	50.42 ± 0.13	50.27 ± 0.00	55.87 ± 0.10	10.74 ± 0.02	10.67 ± 0.01	10.98 ± 0.01
**C18:3**	0.08 ± 0.00	0.08 ± 0.00	0.07 ± 0.01	0.62 ± 0.01	0.61 ± 0.00	0.59 ± 0.01
**C20:0**	0.24 ± 0.00	0.25 ± 0.01	0.23 ± 0.01	0.39 ± 0.01	0.40 ± 0.01	0.42 ± 0.00
**C20:1**	0.15 ± 0.00	0.16 ± 0.01	0.14 ± 0.00	0.32 ± 0.00	0.31 ± 0.00	0.32 ± 0.00
**C22:0**	0.70 ± 0.00	0.71 ± 0.00	0.67 ± 0.01	0.17 ± 0.01	0.18 ± 0.02	0.18 ± 0.00
**C18:1t**	nd	nd	nd	0.22 ± 0.00	0.21 ± 0.00	0.22 ± 0.00
**C18:2t**	0.34 ± 0.01	0.34 ± 0.00	0.32 ± 0.01	0.12 ± 0.01	0.14 ± 0.08	0.15 ± 0.01
**C18:3t**	nd	nd	nd	0.04 ± 0.00	nd	0.04 ± 0.00
**ΣAG *trans***	0.34 ± 0.01	0.34 ± 0.00	0.36 ± 0.01	0.17 ± 0.01	0.35 ± 0.02	0.41 ± 0.01

SO, sunflower oil; OPO, olive pomace oil; OPO25, a blend of SO and OPO containing 25 wt% OPO; OPO50, a blend of SO and OPO containing 50 wt% OPO; nd, not detected. Results represent the mean followed by the standard deviation of 3 analytical determinations (n=3).

Table S2 Fatty acid compositions (%) of the oil blends.

	OPO25-1	OPO25-2	OPO25-3	OPO50-1	OPO50-2	OPO50-3
**C16:0**	7.81 ± 0.09	7.62 ± 0.03	8.02 ± 0.16	9.35 ± 0.12	9.10 ± 0.10	9.39 ± 0.04
**C16:1**	0.31 ± 0.01	0.30 ± 0.00	0.34 ± 0.01	0.50 ± 0.01	0.50 ± 0.01	0.55 ± 0.00
**C18:0**	3.34 ± 0.01	3.33 ± 0.01	3.29 ± 0.01	3.05 ± 0.01	3.08 ± 0.00	2.99 ± 0.01
**C18:1**	46.42 ± 0.17	46.08 ± 0.07	42.04 ± 0.25	55.50 ± 0.35	54.75 ± 0.36	51.42 ± 0.10
**C18:2**	39.97 ± 0.28	40.54 ± 0.09	44.51 ± 0.37	29.45 ± 0.52	30.52 ± 0.37	33.70 ± 0.14
**C18:3**	0.24 ± 0.01	0.23 ± 0.01	0.22 ± 0.01	0.38 ± 0.01	0.35 ± 0.01	0.33 ± 0.00
**C20:0**	0.31 ± 0.02	0.33 ± 0.00	0.30 ± 0.02	0.38 ± 0.01	0.38 ± 0.00	0.36 ± 0.00
**C20:1**	0.20 ± 0.00	0.20 ± 0.00	0.18 ± 0.00	0.23 ± 0.00	0.22 ± 0.01	0.22 ± 0.00
**C22:0**	0.58 ± 0.02	0.60 ± 0.00	0.55 ± 0.03	0.40 ± 0.02	0.71 ± 0.00	0.67 ± 0.01
**C18:1t**	0.13 ± 0.02	0.10 ± 0.00	0.06 ± 0.06	0.19 ± 0.03	0.14 ± 0.02	0.13 ± 0.01
**C18:2t**	0.28 ± 0.03	0.29 ± 0.01	0.29 ± 0.01	0.23 ± 0.01	0.23 ± 0.00	0.23 ± 0.01
**C18:3t**	nd	nd	nd	0.01 ± 0.01	0.01 ± 0.01	0.02 ± 0.00
**ΣAG *trans***	0.41 ± 0.04	0.40 ± 0.01	0.35 ± 0.05	0.42 ± 0.02	0.39 ± 0.01	0.37 ± 0.01

nd, not detected. Results represent the mean followed by the standard deviation of 3 analytical determinations (n=3).

Table S3 Quality parameters of the oils.

	SO-1	SO-2	SO-3	OPO-1	OPO-2	OPO-3
**Acidity (%)**	0.11<br>± 0.000	0.11<br>± 0.000	0.08<br>± 0.002	0.08<br>± 0.000	0.08<br>± 0.000	0.07<br>± 0.000
**PV (meq/kg)**	3.58<br>± 0.06	3.29<br>± 0.03	10.7<br>± 0.1	3.79<br>± 0.03	2.97<br>± 0.04	1.86<br>± 0.05
**OSI (h)**	5.48<br>± 0.05	5.65<br>± 0.08	4.62<br>± 0.05	18.0<br>± 0.9	14.9<br>± 0.4	14.9<br>± 0.7

PV, peroxide value; OSI, oxidative stability index determined in the Rancimat test at 110 °C. Results represent the mean and the standard deviation of 3 analytical determinations (n=3).

Table S4 Quality parameters of the oil blends.

	OPO25-1	OPO25-2	OPO25-3	OPO50-1	OPO50-2	OPO50-3
**Acidity (%)**	0.09<br>± 0.000	0.09<br>± 0.000	0.07<br>± 0.000	0.10<br>± 0.000	0.10<br>± 0.000	0.08<br>± 0.000
**PV (meq/kg)**	3.74<br>± 0.03	3.05<br>± 0.03	4.06<br>± 0.03	3.69<br>± 0.04	3.13<br>± 0.02	6.26<br>± 0.04
**OSI (h)**	6.67<br>± 0.09	6.98<br>± 0.13	6.04<br>± 0.10	8.87<br>± 0.16	9.10<br>± 0.09	7.95<br>± 0.11

PV, peroxide value; OSI, oxidative stability index determined in the Rancimat test at 110 °C. Results represent the mean and the standard deviation of 3 analytical determinations (n=3).

Table S5 Influence of the beating process on the peroxide value (meq/kg fat).

Sample	Oil	Batter
**SO-1**	3.58 ± 0.06a	80 ± 2b
**SO-2**	3.29 ± 0.03a	94 ± 1b
**SO-3**	10.7 ± 0.1a	100 ± 2b
**OPO25-1**	3.74 ± 0.03a	85 ± 1b
**OPO25-2**	3.05 ± 0.03a	80 ± 1b
**OPO25-3**	4.06 ± 0.03a	81 ± 1b
**OPO50-1**	3.69 ± 0.04a	92 ± 7b
**OPO50-2**	3.13 ± 0.02a	81 ± 3b
**OPO50-3**	6.26 ± 0.04a	98 ± 1b
**OPO-1**	3.79 ± 0.03a	95 ± 1b
**OPO-2**	2.97 ± 0.04a	7.4 ± 0.1b
**OPO-3**	1.86 ± 0.05a	10.3 ± 0.3b

Results represent the mean value followed by the standard deviation of 3 analytical determinations. Different letters indicate significant differences according to Student's *t*-test ($p < 0.05$).

Table S6 Influence of the beating process on the levels of fat components (g/100 g fat) differing in molecular weight by direct HPSEC analysis in cupcakes prepared with sunflower oil (SO) or olive pomace oil (OPO).

**Sample**		**TGD**	**DG***	**MG**	**FFA**
**SO-1**	Oil	0.55 ± 0.03a	1.70 ± 0.17a	0.12 ± 0.01a	0.28 ± 0.02a
	Batter	0.60 ± 0.03a	1.73 ± 0.02a	0.22 ± 0.02b	0.58 ± 0.01b
**SO-2**	Oil	0.53 ± 0.03a	1.66 ± 0.17a	0.12 ± 0.01a	0.28 ± 0.02a
	Batter	0.56 ± 0.01a	1.45 ± 0.15a	0.22 ± 0.02b	0.45 ± 0.01b
**SO-3**	Oil	0.99 ± 0.05b	1.54 ± 0.16a	0.14 ± 0.01a	0.34 ± 0.02a
	Batter	0.91 ± 0.01a	1.48 ± 0.03a	0.17 ± 0.01b	0.45 ± 0.01b
**OPO-1**	Oil	0.94 ± 0.05a	6.92 ± 0.71a	0.51 ± 0.05a	0.47 ± 0.03a
	Batter	1.05 ± 0.032a	6.42 ± 0.15a	0.66 ± 0.02b	0.95 ± 0.05b
**OPO-2**	Oil	0.85 ± 0.04a	7.17 ± 0.73a	0.57 ± 0.06a	0.57 ± 0.03a
	Batter	0.91 ± 0.03a	6.96 ± 0.01a	0.71 ± 0.02b	0.86 ± 0.03b
**OPO-3**	Oil	1.10 ± 0.06a	7.79 ± 0.79a	0.65 ± 0.06a	0.66 ± 0.04a
	Batter	1.01 ± 0.01a	7.53 ± 0.10a	0.68 ± 0.01a	0.80 ± 0.01b

*Chromatographic peak partially resolved. TGD, triglyceride dimers; DG, diglycerides; MG, monoglycerides; FFA, free fatty acids and other oil minor components. Results represent the mean value followed by the standard deviation of 3 analytical determinations. Different letters for a given group of compounds indicate significant differences between oil and batter according to Student's *t*-test ($p < 0.05$).

Table S7 Influence of the baking process on the peroxide value (meq/kg fat).

Sample	Batter	Cupcake
**SO-1**	80 ± 2a	84 ± 4a
**SO-2**	94 ± 1a	113 ± 2b
**SO-3**	100 ± 2b	89 ± 2a
**OPO25-1**	85 ± 1a	88 ± 3a
**OPO25-2**	80 ± 1a	77 ± 3a
**OPO25-3**	81 ± 1a	78 ± 3a
**OPO50-1**	92 ± 7b	83 ± 1a
**OPO50-2**	81 ± 3a	81 ± 3a
**OPO50-3**	98 ± 1b	83 ± 1a
**OPO-1**	95 ± 1b	80 ± 6a
**OPO-2**	7.4 ± 0.1a	39 ± 3b
**OPO-3**	10.3 ± 0.3a	77 ± 3b

Results represent the mean value followed by the standard deviation of 3 analytical determinations in an only oil extract (Batter) or in oil extracts of 3 independent samples (Cupcake). Different letters in a row indicate significant differences according to Student's *t*-test ($p < 0.05$).

Table S8 Changes in the content of squalene (mg/kg fat extract) and phytosterols (mg/kg fat extract) at the end of the shelf life (6 months).

Sample	Squalene		Total Phytosterols	
	Initial	Final	Initial	Final
**Girasol 1**	76 ± 11a	72 ± 1a	2346 ± 80a	2489 ± 116a
**Orujo 25-1**	359 ± 12b	400 ± 48a	2628 ± 155a	2991 ± 225a
**Orujo 50-1**	621 ± 8a	657 ± 32a	2370 ± 154a	2406 ± 84a
**Orujo 1**	1286 ± 52a	1337 ± 41a	2448 ± 121a	2491 ± 122a

Results represent the mean value followed by the standard deviation of 3 independent samples. Different letters indicate significant differences between the initial and final contents according to Student's *t*-test ($p < 0.05$).

Table S9 Changes in the content of triterpenic compounds (mg/kg fat extract) in OPO-1 at the end of the shelf-life (6 months).

	**Initial**	**Final**
**Triterpenic alcohols**	610 ± 21a	597 ± 16a
Erythrodiol	532 ± 17a	527 ± 24a
Uvaol	78 ± 6a	70 ± 8a
**Triterpenic acids**	66 ± 9a	71 ± 11a
Oleanolic	54 ± 6a	58 ± 8a
Ursolic	12 ± 3a	13 ± 4a
Maslinic	nd	nd

nd, not detected. Results represent the mean value followed by the standard deviation of 3 independent samples. Different letters indicate significant differences between the initial and final contents according to Student's *t*-test ($p < 0.05$).

Table S10 Consumer panel results for different attributes of cupcakes stored for 3 months.

Attributes	Samples				ANOVA / *p*-value
	SO-1	OPO25-1	OPO50-1	OPO-1	
**Global appraisal**	5.8 ± 1.9a	5.7 ± 1.9a	6.1 ± 1.6a	6.1 ± 1.7a	0.373
**Appearance**	6.4 ± 1.8a	6.2 ± 1.8a	6.2 ± 1.6a	6.3 ± 1.7a	0.971
**Fresh appearance**	5.5 ± 1.8a	5.4 ± 1.8a	5.6 ± 1.6a	5.6 ± 1.8a	0.953
**Colour**	6.5 ± 1.5a	6.4 ± 1.7a	6.6 ± 1.5a	6.6 ± 1.7a	0.885
**Aroma**	5.7 ± 1.8a	5.9 ± 1.8a	6.0 ± 1.7a	6.1 ± 1.9a	0.613
**Flavour**	5.5 ± 1.9a	5.7 ± 2.0a	6.2 ± 1.7a	6.2 ± 1.7a	0.110
**Taste Intensity***	2.9 ± 0.9a	2.8 ± 0.8a	2.8 ± 0.8a	2.9 ± 0.7a	0.743
**Sweet Taste***	2.9 ± 0.8a	2.8 ± 0.7a	3.0 ± 0.7a	3.0 ± 0.8a	0.373
**Texture**	4.5 ± 2.1a	4.4 ± 2.0a	4.8 ± 1.9a	4.7 ± 2.0a	0.651
**Sponginess**	3.8 ± 1.9a	3.7 ± 2.3a	4.3 ± 2.0a	4.0 ± 2.2a	0.386
**Hard-soft texture**	3.9 ± 1.7a	4.0 ± 1.9a	4.1 ± 1.8a	4.1 ± 1.9a	0.900
**Dry-juicy texture**	3.7 ± 1.8a	3.8 ± 2.0a	4.0 ± 1.9a	3.9 ± 2.0a	0.867
**Freshness in mouth**	4.4 ± 2.0a	4.2 ± 2.1a	4.5 ± 1.8a	4.6 ± 2.0a	0.724

Results represent the mean value followed by the standard deviation of 61 assessments (n=61). The attributes were assessed using a structured 9-level hedonic scale in which level 1 stands for "I do not like it at all" and level 9 "I like it very much". Those attributes marked with an asterisk were assessed on a structured 5-level hedonic scale in which level 1 stands for "much milder than I would like" or "much less sweet than I would like", level 2 for "a little milder or a little less sweet than I would like", level 3 for "just as I like", level 4 for "a little more intense or a little sweeter than I would like" and level 5 for "much more intense or much sweeter than I would like". Different letters indicate significant differences between samples according to Duncan's test ($p < 0.05$).

Table S11 Consumer panel results for different attributes of cupcakes stored for 6 months.

Attributes	Samples				ANOVA / *p*-value
	SO-1	OPO25-1	OPO50-1	OPO-1	
**Global appraisal**	5.7 ± 1.8a	5.9 ± 1.8a	5.6 ± 2.0a	5.9 ± 2.0a	0.811
**Appearance**	6.4 ± 1.7a	6.5 ± 1.9a	6.1 ± 2.0a	6.5 ± 1.8a	0.618
**Fresh appearance**	5.6 ± 2.0a	5.6 ± 2.1a	5.4 ± 2.2a	5.6 ± 2.1a	0.879
**Colour**	6.7 ± 1.8a	6.6 ± 1.9a	6.4 ± 2.0a	6.7 ± 1.8a	0.730
**Aroma**	5.7 ± 1.9a	5.7 ± 2.0a	5.2 ± 1.9a	5.9 ± 1.8a	0.275
**Flavour**	5.8 ± 1.9a	6.0 ± 2.1a	5.5 ± 2.2a	5.9 ± 1.9a	0.606
**Taste Intensity***	2.8 ± 0.8a	2.7 ± 0.7a	2.9 ± 0.8a	2.8 ± 0.9a	0.691
**Sweet Taste***	2.9 ± 0.8a	3.0 ± 0.7a	2.9 ± 0.8a	2.9 ± 0.8a	0.950
**Texture**	4.4 ± 2.0a	4.5 ± 2.0a	4.2 ± 2.1a	4.4 ± 2.1a	0.929
**Sponginess**	3.6 ± 2.0a	4.0 ± 2.1a	3.7 ± 2.1a	3.8 ± 2.0a	0.747
**Hard-soft texture**	3.9 ± 1.9a	4.0 ± 2.1a	3.5 ± 2.0a	4.0 ± 2.0a	0.547
**Dry-juicy texture**	3.7 ± 1.9a	3.9 ± 2.1a	3.4 ± 2.2a	3.9 ± 2.0a	0.641
**Freshness in mouth**	4.3 ± 2.1a	4.4 ± 2.2a	4.0 ± 2.3a	4.2 ± 2.0a	0.658

Results represent the mean value followed by the standard deviation of 61 assessments (n=61). The attributes were assessed using a structured 9-level hedonic scale in which level 1 stands for "I do not like it at all" and level 9 "I like it very much". Those attributes marked with an asterisk were assessed on a structured 5-level hedonic scale in which level 1 stands for "much milder than I would like" or "much less sweet than I would like", level 2 for "a little milder or a little less sweet than I would like", level 3 for "just as I like", level 4 for "a little more intense or a little sweeter than I would like" and level 5 for "much more intense or much sweeter than I would like". Different letters indicate significant differences between samples according to Duncan's test ($p < 0.05$).

Table S12 Consumer panel results for different attributes of cupcakes containing SO-1 over storage.

Attributes	Samples			ANOVA / *p*-value
	0-M	3-M	6-M	
**Global appraisal**	6.5 ± 1.5a	5.8 ± 1.9b	5.7 ± 1.8b	0.027
**Appearance**	6.9 ± 1.4a	6.4 ± 1.8a	6.4 ± 1.7a	0.137
**Fresh appearance**	6.6 ± 1.6a	5.5 ± 1.8b	5.6 ± 2.0b	< 0.001
**Colour**	6.7 ± 1.6a	6.5 ± 1.5a	6.7 ± 1.8a	0.684
**Aroma**	6.7 ± 1.7a	5.7 ± 1.8b	5.7 ± 1.9b	0.002
**Flavour**	6.3 ± 1.6a	5.5 ± 1.9b	5.8 ± 1.9ab	0.043
**Taste Intensity***	2.7 ± 0.7a	2.9 ± 0.9a	2.8 ± 0.8a	0.341
**Sweet Taste***	2.9 ± 0.7a	2.9 ± 0.8a	2.9 ± 0.8a	0.897
**Texture**	5.8 ± 1.6a	4.5 ± 2.1b	4.4 ± 2.0b	< 0.001
**Sponginess**	5.5 ± 1.9a	3.8 ± 1.9b	3.6 ± 2.0b	< 0.001
**Hard-soft texture**	5.3 ± 1.6a	3.9 ± 1.7b	3.9 ± 1.9b	< 0.001
**Dry-juicy texture**	5.1 ± 1.6a	3.7 ± 1.8b	3.7 ± 1.9b	< 0.001
**Freshness in mouth**	5.7 ± 1.8a	4.4 ± 2.0b	4.3 ± 2.1b	< 0.001

Results represent the mean value followed by the standard deviation of 61 assessments (n=61). The attributes were assessed using a structured 9-level hedonic scale in which level 1 stands for "I do not like it at all" and level 9 "I like it very much". Those attributes marked with an asterisk were assessed on a structured 5-level hedonic scale in which level 1 stands for "much milder than I would like" or "much less sweet than I would like", level 2 for "a little milder or a little less sweet than I would like", level 3 for "just as I like", level 4 for "a little more intense or a little sweeter than I would like" and level 5 for "much more intense or much sweeter than I would like". Different letters indicate significant differences between samples according to Duncan's test ($p < 0.05$).

Table S13 Consumer panel results for different attributes of cupcakes containing OPO25-1 over storage.

Attributes	Samples			ANOVA / *p*-value
	0-M	3-M	6-M	
**Global appraisal**	6.7 ± 1.3a	5.7 ± 1.9b	5.9 ± 1.8b	0.002
**Appearance**	7.0 ± 1.3a	6.2 ± 1.8b	6.5 ± 1.9ab	0.067
**Fresh appearance**	6.7 ± 1.3a	5.4 ± 1.8b	5.6 ± 2.1b	< 0.001
**Colour**	6.9 ± 1.5a	6.4 ± 1.7a	6.6 ± 1.9a	0.281
**Aroma**	6.7 ± 1.5a	5.9 ± 1.8b	5.7 ± 2.0b	0.005
**Flavour**	6.9 ± 1.3a	5.7 ± 2.0b	6.0 ± 2.1b	0.002
**Taste Intensity***	2.8 ± 0.7a	2.8 ± 0.8a	2.7 ± 0.7a	0.916
**Sweet Taste***	3.0 ± 0.6a	2.8 ± 0.7a	3.0 ± 0.7a	0.254
**Texture**	6.0 ± 1.6a	4.4 ± 2.0b	4.5 ± 2.0b	< 0.001
**Sponginess**	5.8 ± 1.6a	3.7 ± 2.3b	4.0 ± 2.1b	< 0.001
**Hard-soft texture**	5.7 ± 1.5a	4.0 ± 1.9b	4.0 ± 2.1b	< 0.001
**Dry-juicy texture**	5.6 ± 1.7a	3.8 ± 2.0b	3.9 ± 2.1b	< 0.001
**Freshness in mouth**	5.9 ± 1.4a	4.2 ± 2.1b	4.4 ± 2.2b	< 0.001

Results represent the mean value followed by the standard deviation of 61 assessments (n=61). The attributes were assessed using a structured 9-level hedonic scale in which level 1 stands for “I do not like it at all” and level 9 “I like it very much”. Those attributes marked with an asterisk were assessed on a structured 5-level hedonic scale in which level 1 stands for “much milder than I would like” or “much less sweet than I would like”, level 2 for “a little milder or a little less sweet than I would like”, level 3 for “just as I like”, level 4 for “a little more intense or a little sweeter than I would like” and level 5 for “much more intense or much sweeter than I would like”. Different letters indicate significant differences between samples according to Duncan’s test ($p < 0.05$).

Table S14 Consumer panel results for different attributes of cupcakes containing OPO50-1 over storage.

Attributes	Samples			ANOVA / *p*-value
	**0-M**	**3-M**	**6-M**	
**Global appraisal**	6.5 ± 1.3a	6.1 ± 1.6ab	5.6 ± 2.0b	0.012
**Appearance**	6.9 ± 1.5a	6.2 ± 1.6b	6.1 ± 2.0b	0.030
**Fresh appearance**	6.7 ± 1.3a	5.6 ± 1.6b	5.4 ± 2.2b	< 0.001
**Colour**	7.0 ± 1.4a	6.6 ± 1.5ab	6.4 ± 2.0b	0.106
**Aroma**	6.7 ± 1.7a	6.0 ± 1.7b	5.2 ± 1.9c	< 0.001
**Flavour**	6.8 ± 1.4a	6.2 ± 1.7a	5.5 ± 2.2b	< 0.001
**Taste Intensity***	2.7 ± 0.7a	2.8 ± 0.8a	2.9 ± 0.8a	0.286
**Sweet Taste***	3.0 ± 0.6a	3.0 ± 0.7a	2.9 ± 0.8a	0.561
**Texture**	5.8 ± 1.5a	4.8 ± 1.9b	4.2 ± 2.1b	< 0.001
**Sponginess**	5.4 ± 1.7a	4.3 ± 2.0b	3.7 ± 2.1b	< 0.001
**Hard-soft texture**	5.5 ± 1.4a	4.1 ± 1.8b	3.5 ± 2.0b	< 0.001
**Dry-juicy texture**	5.2 ± 1.5a	4.0 ± 1.9b	3.4 ± 2.2b	< 0.001
**Freshness in mouth**	5.9 ± 1.4a	4.5 ± 1.8b	4.0 ± 2.3b	< 0.001

Results represent the mean value followed by the standard deviation of 61 assessments (n=61). The attributes were assessed using a structured 9-level hedonic scale in which level 1 stands for "I do not like it at all" and level 9 "I like it very much". Those attributes marked with an asterisk were assessed on a structured 5-level hedonic scale in which level 1 stands for "much milder than I would like" or "much less sweet than I would like", level 2 for "a little milder or a little less sweet than I would like", level 3 for "just as I like", level 4 for "a little more intense or a little sweeter than I would like" and level 5 for "much more intense or much sweeter than I would like". Different letters indicate significant differences between samples according to Duncan's test ($p < 0.05$).

Table S15 Consumer panel results for different attributes of cupcakes containing OPO-1 over storage.

**Attributes**	**Samples**			**ANOVA / *p*-value**
	**0-M**	**3-M**	**6-M**	
**Global appraisal**	6.6 ± 1.4a	6.1 ± 1.7ab	5.9 ± 2.0b	0.052
**Appearance**	6.7 ± 1.4a	6.3 ± 1.7a	6.5 ± 1.8a	0.391
**Fresh appearance**	6.7 ± 1.3a	5.6 ± 1.8b	5.6 ± 2.1b	< 0.001
**Colour**	7.0 ± 1.4a	6.6 ± 1.7a	6.7 ± 1.8a	0.393
**Aroma**	7.0 ± 1.4a	6.1 ± 1.9b	5.9 ± 1.8b	0.002
**Flavour**	6.8 ± 1.2a	6.2 ± 1.7b	5.9 ± 1.9b	0.011
**Taste Intensity***	2.9 ± 0.7a	2.9 ± 0.7a	2.8 ± 0.9a	0.560
**Sweet Taste***	3.1 ± 0.7a	3.0 ± 0.8a	2.9 ± 0.8a	0.318
**Texture**	6.0 ± 1.7a	4.7 ± 2.0b	4.4 ± 2.1b	< 0.001
**Sponginess**	5.8 ± 2.0a	4.0 ± 2.2b	3.8 ± 2.0b	< 0.001
**Hard-soft texture**	6.0 ± 1.6a	4.1 ± 1.9b	4.0 ± 2.0b	< 0.001
**Dry-juicy texture**	5.5 ± 1.8a	3.9 ± 2.0b	3.9 ± 2.0b	< 0.001
**Freshness in mouth**	6.0 ± 1.6a	4.6 ± 2.0b	4.2 ± 2.0b	< 0.001

Results represent the mean value followed by the standard deviation of 61 assessments (n=61). The attributes were assessed using a structured 9-level hedonic scale in which level 1 stands for "I do not like it at all" and level 9 "I like it very much". Those attributes marked with an asterisk were assessed on a structured 5-level hedonic scale in which level 1 stands for "much milder than I would like" or "much less sweet than I would like", level 2 for "a little milder or a little less sweet than I would like", level 3 for "just as I like", level 4 for "a little more intense or a little sweeter than I would like" and level 5 for "much more intense or much sweeter than I would like". Different letters indicate significant differences between samples according to Duncan's test ($p < 0.05$).
